# Mineral bone disorders and kidney disease in hospitalized children with sickle cell anemia

**DOI:** 10.3389/fped.2022.1078853

**Published:** 2023-02-02

**Authors:** Anthony Batte, Philip Kasirye, Reagan Baluku, Sarah Kiguli, Robert Kalyesubula, Chandy C. John, Andrew L. Schwaderer, Erik A. Imel, Andrea L. Conroy

**Affiliations:** ^1^ Child Health and Development Centre, Makerere University College of Health Sciences, Kampala, Uganda; ^2^ Department of Paediatrics and Child Health, Makerere University College of Health Sciences, Kampala, Uganda; ^3^ Department of Physiology, Makerere University College of Health Sciences, Kampala, Uganda; ^4^ Department of Pediatrics, Indiana University School of Medicine, Indianapolis, IN, United States; ^5^ Ryan White Center for Pediatric Infectious Diseases and Global Health, Indianapolis, IN, United States

**Keywords:** mineral bone disease, acute kidney injury, sickle cell anemia (SCA), mortality, pediatrics, acute kidney disease (AKD)

## Abstract

**Background:**

Mineral bone disorders (MBD) are common in sickle cell anemia (SCA). Frequent vaso-occlusive crises (VOC) further impact MBD in children with SCA. We evaluated the prevalence of markers of SCA-related MBD (sMBD) in hospitalized children and assessed the relationship between sMBD and individual mineral abnormalities with kidney disease.

**Methods:**

We prospectively recruited 185 children with SCA hospitalized with a VOC. Serum measures of mineral bone metabolism (calcium, phosphate, parathyroid hormone, 25-hydroxy vitamin D, FGF23, osteopontin) were measured at enrollment. The primary outcome was markers of sMBD defined as a composite of hypocalcemia, hyperphosphatemia, hyperparathyroidism, or deficiency in 25-OH vitamin D. Secondary outcomes included individual abnormalities in mineral metabolism. The Kidney Disease: Improving Global Outcomes (KDIGO) guidelines were used to define SCA-associated acute kidney injury (AKI). AKI was further assessed using urine NGAL as a marker of tubular injury. Acute kidney disease (AKD) was defined as a composite of AKI, an eGFR < 90 ml/min per 1.73 m^2^ using the Cystatin C GFR equation, or evidence of structural injury (positive biomarker test or albuminuria).

**Results:**

The mean age of children was 8.9 years and 41.6% were female. The prevalence of sMBD was 47.6%, with hypocalcemia the most frequent abnormality (29.9%, 55/184) followed by hyperphosphatemia (20.7%, 38/184), hyperparathyroidism (8.7%, 16/185), and vitamin D deficiency (5.4%, 10/185). There was no association between sMBD and sKDIGO-defined AKI using serial changes in creatinine or when incorporating biomarkers to define AKI. However, the presence of AKD was associated with a 2.01-fold increased odds of sMBD (95% CI 1.05 to 3.83) and was driven by a decrease in eGFR (OR, 2.90 95% CI: 1.59 to 5.29). When evaluating individual mineral abnormalities, hypocalcemia was associated with AKD and low eGFR while hyperparathyroidism was associated with low eGFR, AKI and structural injury. Vitamin D deficiency was associated with structural kidney injury. Vitamin D deficiency, hyperparathryoidism, and increases in FGF23 and osteopontin predicted mortality (*p* < 0.05 for all).

**Conclusion:**

MBD is common among children with SCA hospitalized with VOC. Biomarkers of kidney injury and bone health may help risk stratify children at risk of sMBD. Routine evaluation of sMBD in children with SCA may improve long-term bone health.

## Background

Sickle cell anemia (SCA) is among the commonest inherited hemoglobinopathies and results from a mutation in the *β*-globin gene (*HBB*) (1). The disease is autosomal recessive and characterized by Hemoglobin S polymerization, vaso-occlusion, and hemolysis which in turn leads to ischemia-reperfusion injury, endothelial dysfunction, oxidative stress, and immune activation (1, 2). SCA is a multi-system disease that causes repeated injury and progressive damage to various organs including the brain, kidneys and bones (2). Vaso-occlusive crises are one of the most common complications among children with SCA and are characterized by microvascular occlusions in the bone marrow leading to intense pain in one or more areas of the skeleton (3). Childhood and adolescence is a critical period of bone growth and bone mineral accrual with 90% of bone mass accumulated by 18 years of age (4, 5). Bone mass attained during childhood and adolescence is one of the most important determinants of skeletal health across the lifespan (6).

SCA is associated with growth disturbances and an increased risk of chronic bone problems including chronic pain, avascular necrosis, and vertebral collapse that lead to significant long-term morbidity (3). Mineral bone disorders (MBD) are common in the context of SCA with a number of mineral bone disease abnormalities reported, including hypocalcemia, hyperphosphatemia, hyperparathyroidism, and vitamin D deficiency (7–9). Additional biomarkers that modulate bone mineralization include osteopontin, a phosphorylated glycoprotein that can inhibit bone mineral deposition (10, 11), and fibroblast growth factor-23 (FGF23), a hormone that modulates phosphate handling of the kidneys (12). Acute kidney injury (AKI) develops in up to 36.2% of children with SCA hospitalized with a vaso-occlusive crisis (VOC) (13), and chronic kidney disease (CKD) is reported in up to 26.5% of children with SCA (14). Although MBD is well characterized in CKD (15), there is emerging evidence that MBD occurs during episodes of AKI (16) and may be associated with increased mortality in AKI (17). There are limited data on MBD in children with AKI and SCA thus highlighting the need for more studies.

In this prospective observational study, we evaluated the prevalence of markers of sickle-cell anemia related MBD (sMBD) in children with SCA hospitalized for a painful VOC and assessed the relationship between sMBD and the presence of AKI and other kidney abnormalities.

## Methods

### Study population

Between January and July in 2019, children with sickle cell anemia admitted with a VOC were screened and consecutively enrolled (18). Children were recruited from Mulago National Referral and Teaching Hospital, which is located in Central Uganda and has a high outpatient and inpatient volume. Most children admitted with a VOC are referred from the hospital's dedicated sickle cell clinic that attends to about 1,400 children per year. Routine care includes daily folic acid supplementation, three-monthly malaria prophylaxis with sulfadoxine-pyrimethamine, and penicillin V for children <5 years of age. Hydroxyurea is approved for use and is available in a limited capacity. Care for children with VOC involves hydration using oral or intravenous fluids and analgesic medications based on pain severity and typically includes paracetamol, non-steroidal anti-inflammatory drugs, and opioids (morphine, tramadol).

Eligibility criteria included an age between 2 and 18 years, documented SCA by hemoglobin electrophoresis (hemoglobin SS), hospitalization for a VOC with a pain score ≥2 using an age-specific pain scale. Pain was assessed using the face, legs, activity, cry, and consolability (FLACC) scale (1) in children aged 2–3 years, the Wong-Baker Faces pain scale in children 3–7 years and the numeric pain scale in children ≥ 8 years (2). Children were only eligible to be enrolled once during the recruitment period. The primary outcome was the presence of sMBD defined as a composite of one or more of the following mineral metabolism abnormalities; hypocalcemia, hyperphosphatemia, hyperparathyroidism, or vitamin D deficiency. The individual mineral metabolism abnormalities were secondary outcomes. A sample size of 185 children was generated assuming a prevalence of hypocalcemia of 14% (9). The sample size formula used was $n=\left(z^{2}p\left(1-p\right)/d^{2}\right)$, where; z = standard normal variate corresponding to the 95% confidence interval and is 1.96, d = the required precision of the estimate (0.05), *p* = prevalence rate (19).

### Study procedures

On enrollment all children had a complete history and physical exam to assess medication use, signs of infection, and the site and severity of pain. On admission children had venous blood drawn with serum collected in an additive free tube and allowed to clot at room temperature for 30–60 min before centrifugation at 1200 g for 20 min and storage at −80 °C for subsequent biomarker testing. A spot urine sample was collected using a urine bag or urine container for older children for urinalysis, processing, and storage. Urine samples were spun at room temperature for 5 min at 400 g to remove urine sediment. Urine samples were stored at −80 °C until ELISA testing.

Laboratory tests included complete blood count and serum studies to evaluate biochemical abnormalities in mineral metabolism [Ca^2+^, Mg^2+^, phosphorous, intact parathryroid hormone (iPTH)]. The complete blood counts were measured using the Sysmex automated haematology analyser, version XN 450. Serum laboratory tests were measured by the Johns Hopkins University Infectious Disease Institute Laboratory on serum samples using a Cobas machine (Roche Diagnostics, Indianapolis, USA). Intact PTH was measured in serum by the Ebenezer Clinical Laboratory Ltd. using an electrochemiluminescence immunassay using a Cobas e411 analyzer. Urine albumin and creatinine were tested by the Mulago Hospital Chemistry Laboratory using a Cobas analyzer (Roche Diagnostics, Indianapolis, USA).

### Immunoassays

Additional markers related to bone mineral metabolism and kidney function were measured on stored serum and urine samples by enzyme linked immunoassays (ELISA). Serum cystatin C was measured using the R&D Systems Quantikine ELISA (R&D Systems, Minneapolis, MN) at 1:40 dilution with a reported range of 0.06 to 16 mg/L with pooled serum controls and commercial controls on every plate. The assay has been correlated against the Cystatin C reference standard provided by the Joint Research Centre Institute for Reference Material and Measurements (Catalog # ERM-DA471/ IFCC) with a slope of 1.07 and a R^2^ value of 0.998. Serum FGF23 was measured using a DuoSet by R&D Systems at a 1:5 dilution with a reported range of 200 to 50,000 pg/ml (R&D Systems, Minneapolis, MN). Serum and urine Osteopontin (OPN) were measured using a DuoSet by R&D Systems at a dilution of 1:100 and 1:1,000 respectively (R&D Systems, Minneapolis, MN). Urine NGAL was measured using an ELISA by BioPorto (Kit 036, BioPorto Diagnostics Inc., Hellerup, Denmark) at a 1:1,000 dilution with a reported range of 5–2000 ng/ml according to the manufacturer's protocol. Vitamin D was measured using a commercial assay (Eagle Biosciences Inc, NH, USA) that recognizes total 25-OH vitamin D (including vitamin D3 and D2) in serum with a reported range of 5–600 nmol/L. All sample testing was conducted by trained technicians blinded to patient details. Samples below the limit of detection were assigned the lower limit of the assay.

### Assessment of kidney function

Acute kidney injury (AKI) was defined based on the Kidney Disease: Improving Global Outcomes (KDIGO) guidelines as an increase in serum creatinine of ≥0.3 mg/dl within 48 h or a 50% increase in baseline creatinine within 7 days (3). Kidney function was assessed on enrollment, at 48 h and on day 7 or discharge (whichever happened earlier) using the i-STAT handheld blood analyzer (Abbott Point of Care Inc., Princeton, NJ) with assays traceable to the U.S. National Institute of Standards and Technology (NIST) standard reference material SRM909 with reportable range is 0.20–20.0 mg/dl. For the purposes of defining AKI a creatinine value below the reportable range (<0.20 mg/dl) was assigned a value of 0.19 mg/dl. The participants' lowest creatinine measure was taken as the baseline creatinine. In instances where only a single creatinine measure was available (n = 7), we used the Pottel-age based GFR equation assuming a normal GFR of 120 ml/min per 1.73 m^2^ (20, 21). As previously described, children with an increase in creatinine from 0.2 to 0.3 mg/dl were not considered to have AKI (13).

Defining AKI using creatinine in children with SCA has limitations as the children are likely to have hyperfiltration, increased tubular creatinine excretion (22) and creatinine levels may be affected by reduced muscle mass (23, 24). We thus used the 23rd Acute Disease Quality Initiative (ADQI-23) consensus guidelines which integrate the use of other functional biomarkers of kidney injury in addition to creatinine in defining and staging AKI (25). Based on the ADOQI-23 guidelines, AKI is staged based on presence of a positive or negative kidney injury biomarker on addition to the fold increase in creatinine as defined by the KDIGO guidelines. In our study, a uNGAL level ≥150 ng/ml was considered biomarker positive for tubular injury (BM+) (26). Admission eGFR was calculated using the CKiD cystatin C-based equation, eGFR = 70.69 * (cystatin C)^−0.931^ (27). Acute kidney disease was defined as one or more of the following: AKI or an eGFR < 90 ml/min per 1.73 m^2^ or signs of structural injury (macroalbuminuria or BM+) (25, 28).

### Assessment of mineral bone abnormalities

Bone mineral abnormalities were defined using the following criteria: hypocalcemia (<2.2 mmol/L following correction for serum albumin) (29), hyperphosphatemia (phosphorous > upper limit of normal for age and sex) (30), hyperparathyroid hormone (iPTH > 70 pg/ml) (31), vitamin D deficiency (25-OH vitamin D < 30 nmol/L) (32). A child meeting any of the above criteria was considered to have sickle-cell anemia related MBD (sMBD). In addition, children were classified as having elevated FGF23 and elevated osteopointin if the concentrations exceeded the 95th percentile measured using a population of children with SCA in steady state with the cut-offs as follows: FGF23 > 535 pg/ml and osteopontin >375 ng/ml.

### Statistical analysis

Data were entered into REDCap electronic data capture tools hosted at Indiana University. Data were analyzed using STATA v17.0 (StataCorp) and GraphPad Prism v9. Data are presented descriptively using median and interquartile range (IQR) for continuous variables and the number and frequency for discrete variables. To examine the relationship between continuous variables and dichotomized measures of sMBD, a Wilcoxon rank-sum test was used for continuous variables. For categorical variables, Pearson's Chi-square or Fisher's exact test were used, as appropriate. Spearman's rank correlation was used to evaluate relationships between measures of bone metabolism and kidney function. Logistic regression was used to evaluate the relationship between AKI and AKD with sMBD or individual mineral metabolism abnormalities and models were adjusted for participant age and sex. Bonferroni correction was used to adjust for multiple testing.

## Results

**Figure 1:**
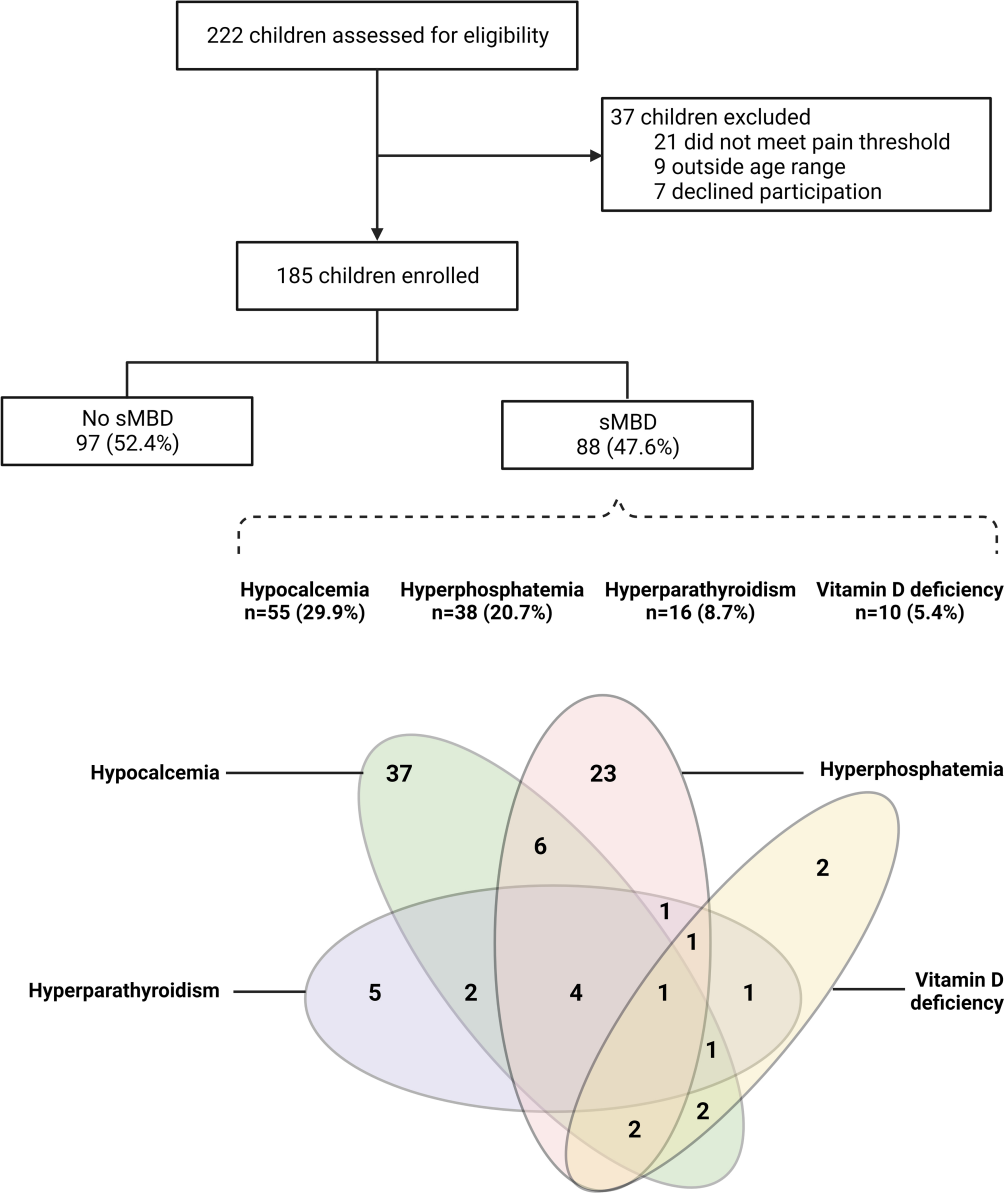
Flowchart of the study population. Flow chart depicting study participants based on sickle cell anemia related mineral bone disorder (sMBD). Below the flowchart is a Venn diagram showing the inter-relationships between parameters used to define sMBD with hypocalcemia (corrected calcium <2.2 mmol/L) present in 55 children (29.9%), hyperphosphatemia (phosphate levels > upper limit of normal for age and sex) present in 38 children (20.7%), hyperparathyroidism (parathyroid hormone >70 pg/ml) present in 16 study participants (8.7%) and Vitamin D deficiency (<30 nmol/L) present in 10 (5.4%) of study participants. Created with BioRender.com.

We assessed sMBD in 185 children with sickle cell anemia hospitalized for a painful VOC as outlined in Figure 1. The median age of children was 8.9 years [interquartile range (IQR), 5.9 to 11.8] and 41.6% of participants were female (Table 1). The median duration of pain prior to presentation was 3 days (IQR, 2 to 4) with a median pain score of 6 (IQR, 4 to 8). Overall, the prevalence of sMBD was 47.6% (Figure 1). Hypocalcemia was the most frequent mineral bone abnormality observed at 29.9% (55/184) followed by hyperphosphatemia at 20.7% (38/184), hyperparathyroidism at 8.7% (16/185) and vitamin D deficiency at 5.4% (10/185). Vitamin D insufficiency (30–50 nmol/L) was present in 24.3% (45/185) of the children.

**Table 1:** Demographic, clinical and laboratory characteristics on admission associated with bone mineral disease.

	Combined (*n* = 185)	No sMBD (*n* = 97)	sMBD (*n* = 88)	*p* value
**Demographic characteristics**				
Age, years, median (IQR)	8.9 (5.8, 11.8)	7.8 (5.0, 11.1)	10.2 (6.7, 13.0)	0.008
Sex, *n* (%) Female	77 (41.6)	41 (42.3)	36 (40.9)	0.851
Height-for-age z score	−1.4 (−2.3, −0.4)	−1.2 (−2.0, −0.3)	−1.5 (−2.7, −0.5)	0.054
Weight-for-age z score	−1.5 (−2.1, −0.5)	−1.6 (−2.2, −0.6)	−1.2 (−1.9, −0.2)	0.243
Weight-for-height z score	−1.6 (−2.2, −0.4)	−1.6 (−2.6, −0.6)	−1.3 (−2.1, 0.3)	0.197
BMI-for-age z score	−1.3 (−2.3, −0.4)	−1.3 (−1.9, −0.4)	−1.2 (−2.7, −0.3)	0.485
Mid Upper Arm Circumference, cm	16 (15.0, 17.8)	16 (14.7, 17.4)	16.5 (15.1, 18.0)	0.111
**Clinical characteristics**				
Temperature, °C	37.1 (36.7, 37.8)	37.1 (36.7, 37.8)	37.1 (36.6, 37.9)	0.830
Heart rate, bpm	108 (98, 121)	108 (98, 121)	110 (95, 124)	0.765
Respiratory rate, bpm	29 (24, 36)	29 (24, 36)	30 (25, 36)	0.764
Blood pressure category, *n* (%)				
Hypotensive	2 (1.1)	0	2 (2.3)	0.306
Normotensive	149 (80.5)	78 (80.4)	71 (80.7)	
Hypertension	34 (18.4)	19 (19.6)	15 (17.1)	
**Pain Assessment**				
Pain Score, median (IQR)	6 (4, 8)	6 (4, 8)	6 (4, 8)	0.290
Duration of pain in days	3 (2, 4)	3 (2, 5)	3 (2, 4)	0.548
**Complete blood count**				
WBC × 10^3^/μl	22.6 (16.7, 33.8)	22.6 (15.5, 33.8)	22.8 (16.7, 36.1)	0.724
Hemoglobin, g/dl	7.2 (6.3, 8.3)	7.3 (6.5, 8.4)	7.1 (6.0, 7.9)	0.188
Platelet count × 10^9^/L	418 (306, 529)	427 (295, 514)	403 (307, 534)	0.913
**Bone mineral measures**				
Ca^2+^, mmol/L	2.2 (2.1, 2.3)	2.3 (2.2, 2.3)	2.2 (2.1, 2.2)	<0.001
PO_4_, mg/dl	5.0 (4.2, 5.7)	4.7 (4.1, 5.4)	5.5 (4.3, 6.5)	<0.001
Mg^2+^, mmol/L	0.9 (0.9, 1.0)	0.9 (0.9, 1.0)	1.0 (0.9, 1.1)	0.023
PTH, pg/ml	30.7 (20.4, 44.7)	24.7 (17.6, 37.1)	35.4 (24.6, 54.7)	<0.001
25-OH Vitamin D, nmol/L	60.3 (46.1, 74.7)	63.6 (51.4, 79.5)	56.5 (40.6, 71.6)	0.003
FGF-23, pg/ml	229 (195, 722)	211 (195, 503)	273 (195, 1139)	0.038
**Kidney function**				
Creatinine, mg/dl	0.30 (0.19, 0.40)	0.30 (0.19, 0.30)	0.30 (0.19, 0.50)	0.032
BUN, mg/dl	4 (3, 7)	3 (3, 5)	4 (3, 8)	<0.001
Sodium, mmol/L	138 (135, 140)	138 (135, 140)	137 (134, 140)	0.271
Potassium, mmol/L	3.8 (3.5, 4.1)	3.7 (3.5, 4.1)	3.8 (3.6, 4.1)	0.389
Cystatin C, mg/L	0.8 (0.6, 1.0)	0.7 (0.6, 0.9)	0.9 (0.7, 1.2)	<0.001
Enrolment eGFR (CKiD, Cystatin C)	86 (68, 107)	94 (76, 118)	78 (61, 100)	<0.0001
Urine NGAL, ng/ml	9.8 (5.0, 33.8)	10.6 (5.0, 38.2)	9.1 (5.0, 27.4)	0.754
**Urinalysis**				
Albuminuria category (adjusted to uCr), *n* (%)				
A1, <3 mg/mmol	105 (60.0)	59 (64.1)	46 (55.4)	0.501
A2, 3–30 mg/mmol	55 (31.4)	26 (28.3)	29 (34.9)	
A3, >30 mg/mmol	15 (8.6)	7 (7.6)	8 (9.6)	

Data presented are median (interquartile range) or *n* (%). Differences in continuous variables were assessed using Wilcoxon rank sum test. Differences in categorical variables were assessed using Pearson's Chi-square test or Fisher's exact test, as appropriate.

### Demographic, clinical, and laboratory characteristics associated with markers of sMBD

Demographic, clinical, and laboratory characteristics are presented in Table 1. Children with sMBD were significantly older than children without sMBD (*p* = 0.008) but were similar in nutritional status and clinical characteristics on admission. There were no differences in the history of pain duration or the severity of pain on admission based on sMBD (*p* > 0.05). In addition to differences in the measures used to define sMBD (Ca^2+^, PO4^−^, PTH, 25-OH vitamin D), children with sMBD had higher levels of Mg^2+^ and FGF23 compared to children without sMBD (*p* < 0.05 for both). There were differences in kidney function (BUN, Cystatin C) in children with sMBD compared to children without sMBD.

**Figure 2:**
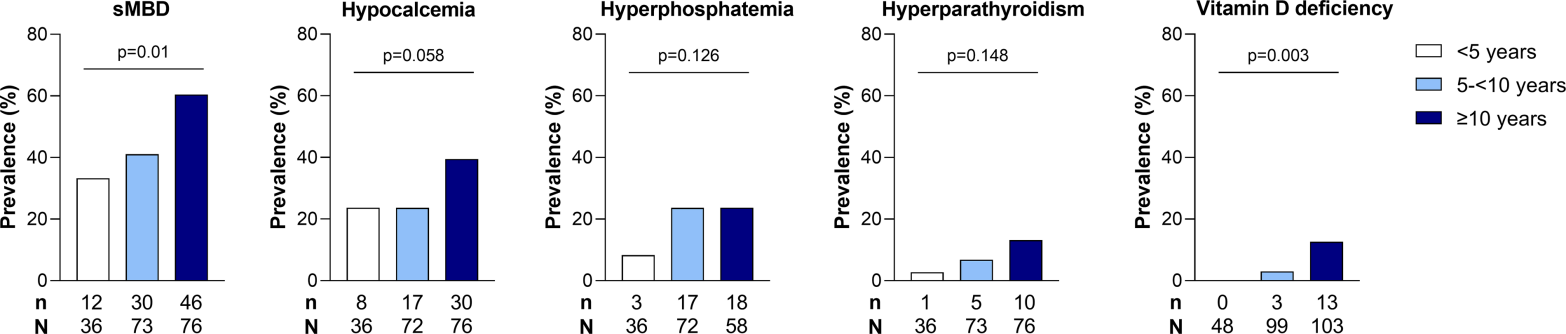
Relationship between age and sMBD in children with SCA. Bar graph depicting the frequency of sMBD prevalence and individual mineral abnormalities across age categories (<5 years, 5- <10 years, ≥10 years). Differences in the frequency of sMBD and mineral abnormalities assessed using Pearson's chi-square test or Fisher's exact test, as appropriate. The *p* values are presented on the bar graph.

We conducted additional analyses evaluating differences in the same measures in children with and without hypocalcemia and hyperphosphatemia as the most frequent findings in children with sMBD (Table 2) and vitamin D deficiency and hyperparathyroidism as the least frequent findings (Table 2). Participants with hypocalcemia, hyperphosphatemia, and hyperparathyroidism all had increased levels of Cystatin C and BUN compared to children with normal values for the same marker of mineral metabolism. Further, children with both vitamin D deficiency or with hyperparathyroidism had higher levels of urine NGAL as a measure of proximal tubular injury, higher potassium, osteopontin, and FGF23 compared to children without vitamin D deficiency or hyperparathyroidsm. When evaluating mineral abnormalities by age, children with hyperparathyroidism and vitamin D deficiency were older (Table 2) and this was further supported by an increase in the frequency of mineral abnormalities across age categories (Figure 2). The earliest abnormality to present was hypocalcemia, present in 22% of children <5 years of age followed by hyperphosphatemia that emerged in children between 5 and 10 years of age, while both hyperparathyroidism and vitamin D deficiency were relatively infrequent before 10 years of age (Figure 2).

**Table 2:** Demographic, clinical and laboratory characteristics on admission associated with mineral bone disorders.

	Calcium			Phosphate		
	No hypocalcemia (*n* = 129)	Hypocalcemia (*n* = 55)	*p* value	No hyperphosphatemia (*n* = 146)	Hyperphosphatemia (*n* = 38)	*p* value
**Demographic characteristics**						
Age, years, median (IQR)	8.2 (5.3, 11.4)	10.3 (7.0, 12.8)	0.068	8.9 (5.3, 11.5)	9.7 (6.7, 13.1)	0.130
Sex, *n* (%) Female	57 (44.2)	19 (34.6)	0.224	60 (41.1)	16 (42.1)	0.910
**Complete blood count**						
WBC × 10^3^/μl	22.4 (16.1, 33.4)	23.0 (16.8, 39.5)	0.597	22.7 (16.4, 33.3)	21.7 (16.7, 40.1)	0.716
Hemoglobin, g/dl	7.5 (6.5, 8.5)	6.4 (5.9, 7.5)	<0.0001	6.9 (6.1, 8.2)	7.6 (6.7, 8.4)	0.037
Haptoglobin, mg/L	29.1 (6.3, 144.3)	19.4 (6.3, 61.8)	0.157	30.4 (6.3, 144.3)	13.6 (6.3, 83.1)	0.199
Platelet count × 10^9^/L	437 (346, 566)	348 (279, 451)	0.003	417 (301, 520)	434 (351, 575)	0.317
**Bone mineral measures**						
Ca^2+^, mmol/L	2.26 (2.21, 2.35)	2.11 (2.05, 2.17)	—	2.21 (2.12, 2.30)	2.23 (2.17, 2.34)	0.224
PO_4_, mg/dl	5.0 (4.2, 5.6)	4.7 (4.1, 6.1)	0.901	4.6 (4.1, 5.4)	6.6 (6.1, 7.2)	—
Mg^2+^, mmol/L	0.9 (0.9, 1.0)	1.0 (0.9, 1.1)	0.123	0.9 (0.9, 1.0)	1.00 (0.9, 1.1)	0.002
PTH, pg/ml	27.0 (18.2, 41.0)	37.0 (25.8, 52.8)	0.001	30.2 (20.4, 42.8)	33.6 (20.8, 52.8)	0.175
25-OH Vitamin D, nmol/L	62.9 (50.5, 74.7)	54.9 (38.6, 76.8)	0.033	61.4 (46.8, 77.9)	57.0 (41.9, 67.7)	0.093
FGF23, pg/ml	217 (195, 623)	314 (195, 1169)	0.065	233 (195, 651)	272 (195, 1610)	0.388
Serum Osteopontin, ng/ml	187 (134, 243)	231 (162, 320)	0.002	199 (144, 254)	208 (132, 334)	0.517
**Kidney function**						
BUN, mg/dl	3 (3, 5)	5 (3, 10)	0.002	3 (3, 6)	5 (3, 9)	0.013
Potassium, mmol/L	3.8 (3.6, 4.1)	3.7 (3.3, 4.1)	0.228	3.7 (3.5, 4.0)	4.0 (3.8, 4.3)	0.001
Cystatin C, mg/L	0.8 (0.6, 1.0)	0.9 (0.7, 1.2)	0.001	0.8 (0.6, 1.0)	1.0 (0.7, 1.3)	0.008
eGFR (CKiD, Cystatin C)	92 (72, 113)	75 (61, 93)	0.001	90 (71, 111)	70 (54, 104)	0.008
Urine NGAL, ng/ml	9.0 (5.0, 30.6)	13.4 (5.0, 38.4)	0.177	10.1 (5.0, 29.6)	8.5 (5.0, 50.0)	0.861

	Parathyroid hormone			Vitamin D		
	No hyperparathyroidism (*n* = 169)	Hyperparathyroidism (*n* = 16)	*p* value	No vitamin D deficiency (*n* = 175)	Vitamin D deficiency (*n* = 10)	*p* value
**Demographic characteristics**						
Age, years, median (IQR)	8.8 (5.4, 11.6)	10.8 (7.9, 13.9)	0.047	8.3 (5.4, 11.5)	12.5 (10.8, 15.3)	<0.001
Sex, n (%) Female	66 (39.1)	11 (68.8)	0.02	98 (41.9)	9 (56.3)	0.261
**Complete blood count**						
WBC × 10^3^/μl	22.5 (16.7, 33.4)	27.1 (16.7, 50.5)	0.536	22.4 (16.7, 33.4)	28.6 (14.2, 98.4)	0.744
Hemoglobin, g/dl	7.3 (6.3, 8.3)	6.7 (6.0, 8.4)	0.620	7.1 (6.3, 8.3)	7.4 (6.4, 8.3)	0.809
Haptoglobin, mg/L	28.7 (6.3, 147.9)	24.2 (6.3, 58.8)	0.239	26.9 (6.3, 144.3)	37.9 (6.3, 72.0)	0.553
Platelet count × 10^9^/L	418 (306, 541)	390 (244, 481)	0.287	418 (306, 533)	349 (304, 538)	0.666
**Bone mineral measures**						
Ca^2+^, mmol/L	2.21 (2.13, 2.32)	2.19 (2.06, 2.29)	0.206	2.2 (2.1, 2.3)	2.2 (2.1, 2.2)	0.047
PO_4_, mg/dl	4.8 (4.1, 5.6)	5.7 (4.9, 6.8)	0.019	5.0 (4.2, 5.6)	6.0 (3.9, 6.9)	0.404
Mg^2+^, mmol/L	0.9 (0.9, 1.0)	1.0 (0.9, 1.3)	0.024	0.9 (0.9, 1.0)	1.0 (0.9, 1.1)	0.212
PTH, pg/ml	27.9 (18.8, 40.2)	102.2 (76.2, 153.7)	—	29.3 (19.1, 42.7)	51.4 (34.0, 147.4)	<0.001
25-OH Vitamin D, nmol/L	61.4 (47.6, 76.7)	40.0 (28.7, 56.7)	<0.001	61.4 (47.8, 76.5)	24.6 (22.7, 26.2)	—
FGF23, pg/ml	223 (195, 558)	4,419 (248, 10350)	<0.001	225 (195, 603)	4,063 (230, 7693)	0.030
Serum Osteopontin, ng/ml	197 (138, 251)	394 (238, 568)	<0.001	202 (143, 259)	243 (198, 441)	0.045
**Kidney function**						
BUN, mg/dl	3 (3, 5)	16 (5, 56)	<0.001	3 (3, 6)	8 (4, 29)	0.057
Potassium, mmol/L	3.8 (3.5, 4.0)	4.2 (3.7, 5.3)	0.014	3.8 (3.5, 4.0)	4.3 (3.9, 5.5)	0.001
Cystatin C, mg/L	0.8 (0.6, 1.0)	1.3 (1.0, 2.4)	<0.001	0.8 (0.6, 1.0)	1.1 (0.8, 1.6)	0.130
eGFR (CKiD, Cystatin C)	90 (70, 111)	54 (31, 70)	<0.001	89 (68, 110)	65 (45, 87)	0.130
Urine NGAL, ng/ml	9.2 (5.0, 27.9)	213.4 (11.3, 1069.8)	<0.001	9.6 (5.0, 29.6)	42.3 (13.7, 983.1)	0.016

Data presented are median (interquartile range) or *n* (%). Differences in continuous variables were assessed using Wilcoxon rank sum test. Differences in categorical variables were assessed using Pearson's Chi-square test.

### Kidney function and its relationship with altered mineral metabolism

**Figure 3:**
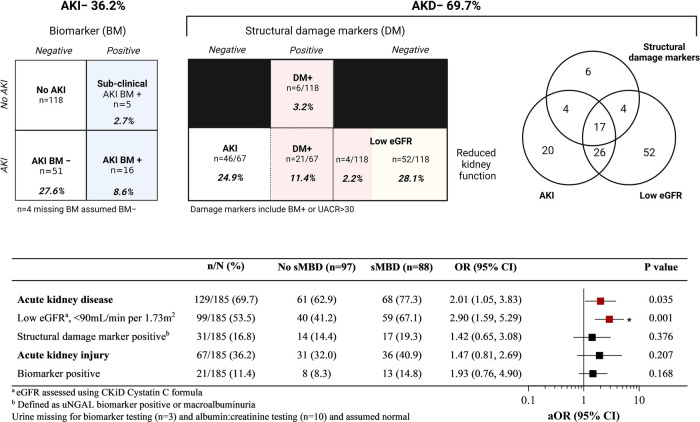
Relationship between acute kidney injury (AKI) and disease (AKD) and mineral bone disease in children with sickle cell anemia hospitalized for a vaso-occlusive crisis. Top. AKI defined using acute changes in creatinine based on sKDIGO definition. AKD was defined based on presence of either a low GFR, AKI or presence of structural damage markers (DM). Structural damage markers included a positive biomarker test (BM+) (urine NGAL ≥ 150 ng/ml), or urine albumin to creatinine ratio (UACR) > 30 mg/mmol. The relationships between measures of kidney injury and disease are presented in the Venn diagram to the right. Bottom. A Forest plot depicting the frequency between different measures of kidney disease and the presence of sMBD. The adjusted odds ratio (aOR) is generated using logistic regression adjusting for participant age and sex. Relationships that were significant (*p* < 0.05) are presented in red and those significant following adjustment for multiple testing are indicated with an asterisk. The adjusted *p* value to account for multiple testing was *p* < 0.01 based on 5 comparisons. Created with BioRender.com.

**Figure 4:**
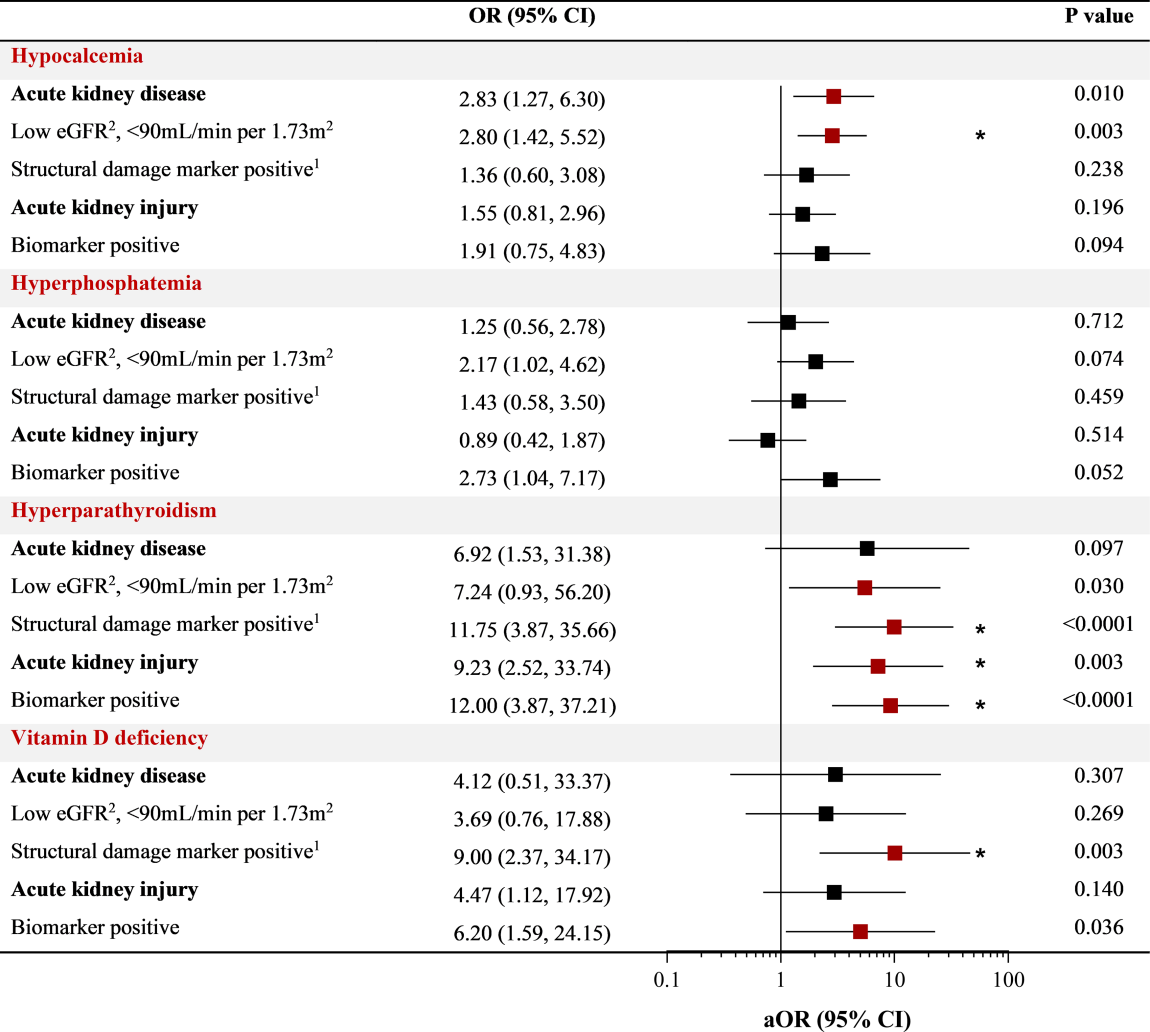
Relationship between kidney function and individual markers of mineral metabolism. Forest plot depicting the odds ratio (OR) and 95% CI between individual abnormalities in bone mineral metabolism (hypocalcemia, hyperphosphatemia, hyperparathyroidism, vitamin D deficiency) and measures of kidney disease using logistic regression. The adjusted OR (aOR) adjusts for participant age and sex with relationships with a *p* < 0.05 in red and an asterisk indicating relationships significant after adjusting for multiple testing at an adjusted alpha of 0.01 within each mineral abnormality category (*).

We further assessed the relationship between kidney function and sMBD based on the presence of AKI and AKD defined during hospitalization. Overall, 36.2% of children had AKI defined based on serial changes in serum creatinine, with 16 children BM + (8.6%) based on a positive uNGAL test, and 69.7% had AKD (Figure 3). There was no difference in sMBD based on the presence of sub-clinical AKI or AKI status (BM− vs. BM+). However, there was an increase in sMBD in children with AKD that encompassed reduced GFR using the Cystatin C based eGFR equation or structural abnormalities including BM + or macroalbuminuria. A low eGFR defined using Cystatin C was also independently associated with sMBD (Figure 3). We further assessed the relationship between individual mineral abnormalities and AKI and AKD. Both BM + AKI and BM− AKI were associated with hyperparathyroidism while BM + AKI was associated with vitamin D deficiency. There was no association between AKI and hypocalcemia or hyperphosphatemia. Children with AKD had increased odds for having hypocalcemia while a low Cystatin C defined eGFR was associated with both hypocalcemia and hyperparathyroidism. Presence of a marker of structural kidney damage was associated with a 9-fold increased odds of having vitamin D deficiency (95% CI 2.37 to 34.17) (Figure 4).

### Relationship between markers of sMBD and mortality

**Figure 5:**
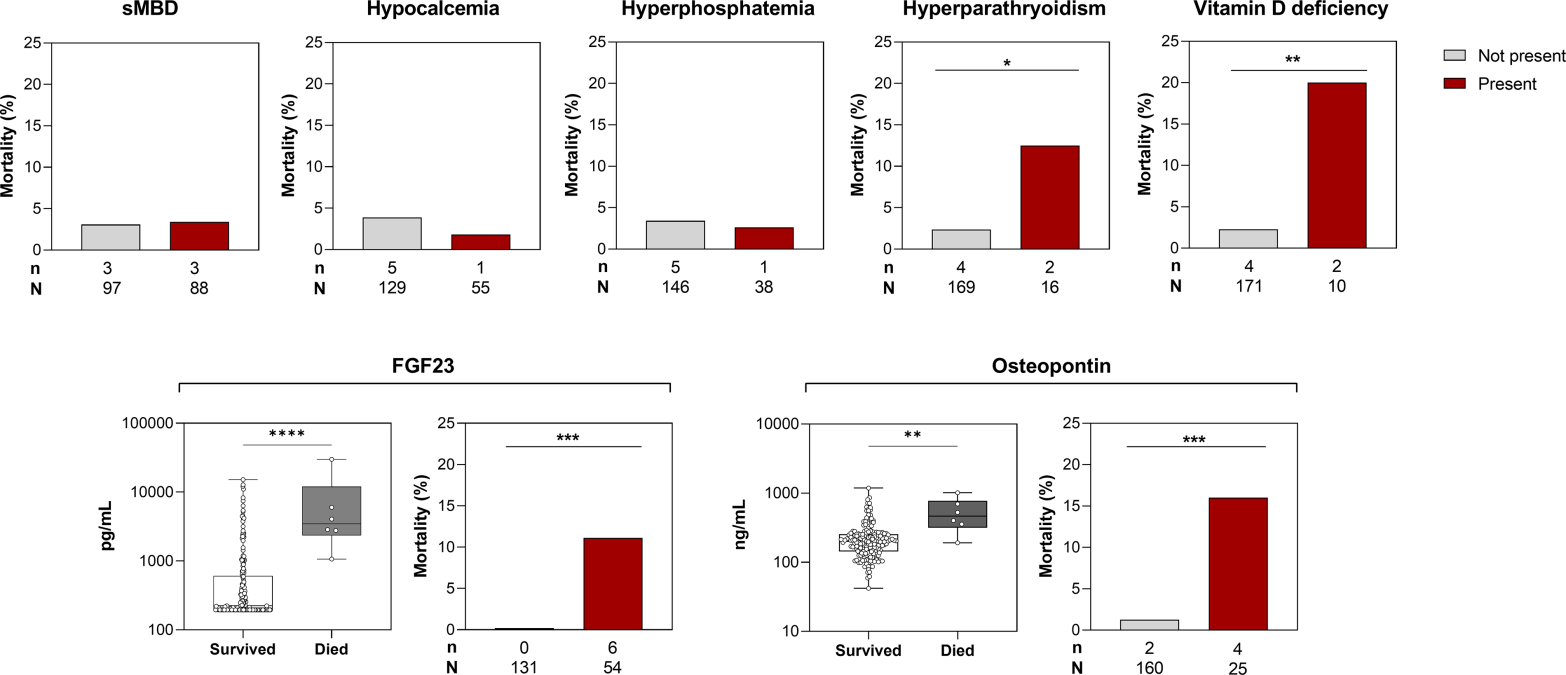
Relationship between sMBD and mortality in children with SCA. Top. Bar graphs depicting the frequency of children who died in-hospital based on the presence of sMBD or individual mineral abnormalities. Bottom. Box plots depicting serum levels of FGF23 and osteopontin at enrollment in children based on in-hospital mortality with differences between groups assessed using the Wilcoxon rank sum test. The corresponding bar graphs depict the frequency of in-hospital death among children with elevated levels of FGF23 and osteopontin using the 95th percentiles from Ugandan children with SCA in steady state to define the reference range. Differences in dichotomous variables and mortality assessed using Fisher's exact test. *p < 0.05, ** *p* < 0.01, ***p < 0.001, and ****p < 0.0001.

In this study, 6/185 (3.2%) of participants died in-hospital with a similar frequency of mortality among children with sMBD 3/88 (3.4%) compared to children without sMBD 3/97 (3.1%). While infrequent, the presence of hyperparathyroidism and vitamin D deficiency were associated with increased mortality (Figure 5). There were significant increases in the median level of serum bone biomarkers FGF23 (*p* < 0.0001) and osteopontin (*p* < 0.01) in children who died in-hospital compared to survivors (*p* < 0.001) (Figure 5). Both elevated FGF23 and osteopontin were associated with higher mortality (*p* < 0.001) (Figure 5).

### Relationship between markers of mineral bone metabolism

**Figure 6:**
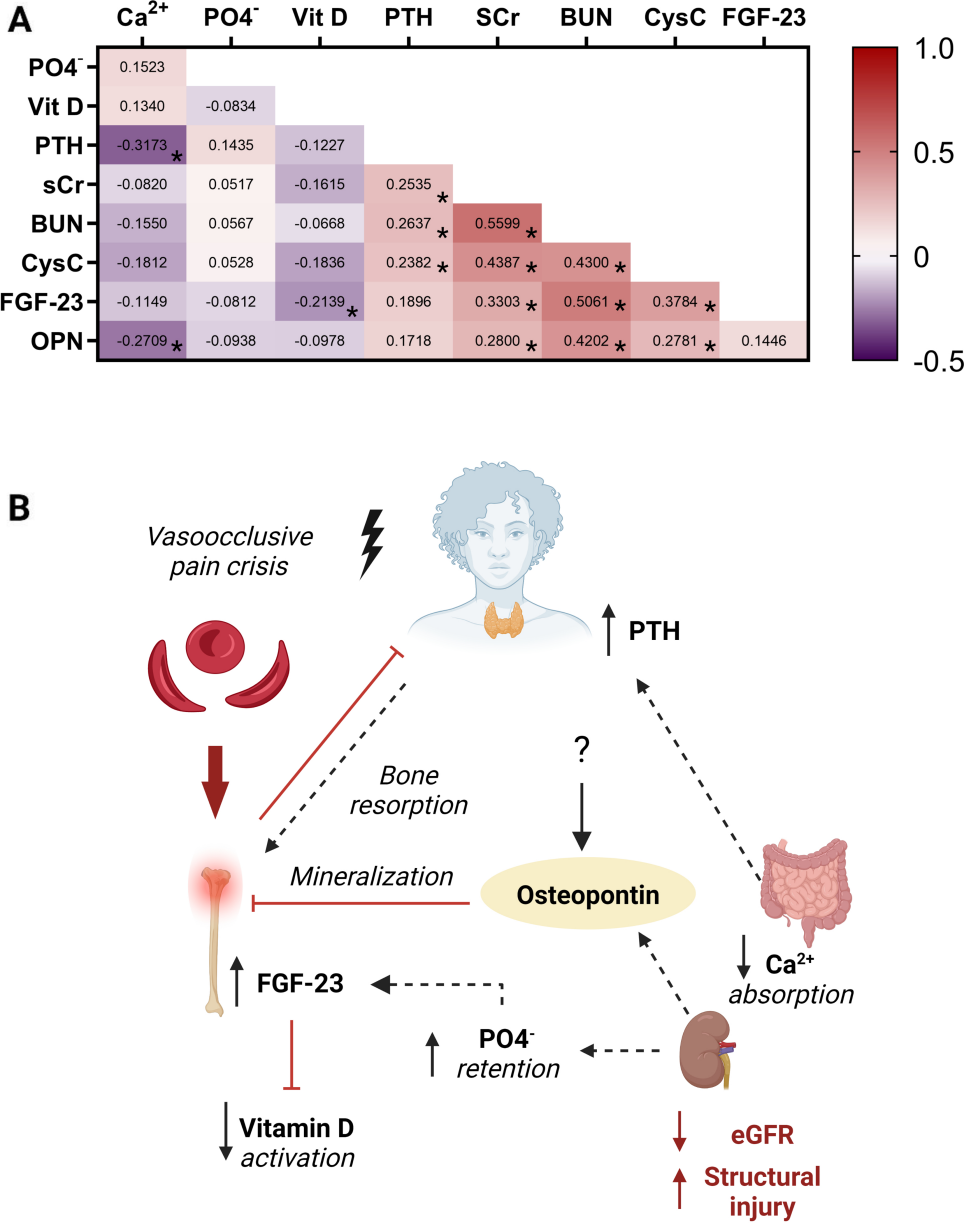
The bone-kidney axis during a vaso-occlusive event. (**A**) Heatmap depicting the Spearman correlation coefficients between serum measures of mineral metabolism and kidney function. Positive relationships are depicted in red while negative relationships are shown in purple. Relationships significant following adjustment for multiple testing are indicated with an asterisk based on an adjusted alpha of 0.005. (**B**) Conceptual diagram depicting the relationship between measures of bone mineral metabolism and kidney function during a vaso-occlusive crisis demonstrating the relationship depicted in the heatmap. Low serum calcium levels and increased phosphate levels lead to hyperparathyroidism with resultant bone resorption. The high phosphate levels due to reduced kidney clearance led to high FGF23 which is known to inhibit vitamin D activation. Osteopontin released by various cells and upregulated in AKI, inhibits bone mineralization. Abbreviations: calcium (Ca^2+^), phosphate (PO4^−^), Vitamin D (Vit D), parathyroid hormone (PTH), serum creatinine (SCr), blood urea nitrogen (BUN), Cystatin C (CysC), fibroblast growth factor 23 (FGF23), Osteopontin (OPN). Created with BioRender.com.

Finally, we conducted correlation analysis to evaluate relationships between individual measures of mineral bone metabolism and kidney function (Figure 6). Within markers of mineral metabolism, parathyroid hormone was positively correlated with phosphate levels and negatively correlated with both calcium and vitamin D levels. FGF23 was negatively correlated with calcium and vitamin D and positively correlated with parathyroid hormone levels, BUN and cystatin C. Parathryroid hormone levels were strongly correlated with markers of reduced kidney filtration (creatinine, BUN, Cystatin C) as well as structural injury to the kidney (urine NGAL) (Figures 4, 6). Osteopontin was negatively correlated with calcium and positively correlated with markers of reduced kidney filtration (creatinine, BUN, Cystatin C) and FGF23.

## Discussion

In this prospective observational study, we demonstrated a high prevalence of sMBD in children hospitalized with a VOC. Hypocalcemia was the most common mineral bone abnormality followed by hyperphosphatemia, hyperparathyroidism and vitamin D deficiency, respectively. With the exception of hyperphosphatemia, all mineral bone abnormalities were associated with kidney disease. sMBD and hypocalcemia were associated with AKD and low eGFR while hyperparathyroidism and vitamin D deficiency were associated with structural injury to the kidney. Although individual features of sMBD varied by age, features associated with mortality during hospitalization (hyperparathryoidism and vitamin D deficiency) were most common in older children. In this population, two additional markers of sMBD, FGF23 and osteopontin, were associated with kidney disease and also strongly associated with mortality.

Mineral bone abnormalities are well known complications of CKD (15, 33); however, there is emerging evidence that AKI is also associated with dysregulation of bone minerals that can impact bone health (16). Data on mineral bone abnormalities in children with AKI and SCA are limited and complicated by challenges in assessing kidney disease in children with SCA due to glomerular hyperfiltration and muscle wasting which affect serum creatinine levels (22–24). In the present study, AKI was assessed using both serum creatinine and urine NGAL as a biomarker of structural kidney injury (25). There were differences in the frequency of sMBD based on AKI status. However, when AKD was evaluated incorporating Cystatin C, a more stable biomarker of glomerular filtration in the context of SCA, and markers of structural kidney injury were incorporated (i.e., macroalbuminuria, positive NGAL), AKD was identified as a risk factor for sMBD. As children with SCA have altered tubular handling of creatinine, alternative biomarkers of kidney function are useful in defining the presence of kidney disease and identifying acute structural injury (34). When defining AKI based on changes in serum creatinine alone (13, 28), children with kidney disease may be misclassified. Thus, the use of a broader AKD definition was able to identify children with AKI and pre-existing kidney disease. As CKD is defined based on a follow-up period of not less than 3 months, a criterion which was not fulfilled in our study, we were unable to differentiate between AKD and CKD.

The most common mineral abnormality in our study was hypocalcemia which occurred in over a quarter of the population and was frequent across the age span. In the context of AKI, low calcium is thought to be secondary to reduced activity of 1 alpha hydroxylase enzyme and vitamin D deficiency (16). Vitamin D deficiency was comparatively uncommon in our cohort, occurring in 5.4% of children although a large proportion of children (24%) had vitamin D insufficiency. A recent multi-country study of 4,509 African children <8 years of age identified vitamin D deficiency in 0.6% of children with seasonal variations in vitamin D levels and increasing vitamin D deficiency in older children (35). Hypocalcemia may also occur as a result of increased intracellular uptake of calcium in sickled cells (36) and as a result of CKD in children with SCA (14, 31). Hypocalcemia and hyperphosphatemia are drivers for elevated parathyroid hormone levels, which were also observed in the population. Parathyroid hormone levels were negatively correlated with serum calcium and positively correlated with serum phosphate levels. The hyperphosphatemia was likely due to reduced renal clearance and was correlated with higher Cystatin C levels as a measure of reduced glomerular filtration in these children (37). In children with sickle cell anemia, hyperphosphatemia may also be secondary to increased tubular reabsorption of phosphorus and resistance to FGF23 (38, 39).

Hyperparathyroidism was associated with multiple measures of kidney function, including structural abnormalities in kidney function and AKI. This suggests that AKI may exacerbate hyperparathyroidism present in SCA (40, 41). Hyperparathyroidism occurred in 8.7% of children with SCA, was more frequent in children ≥10 years of age (13.2%) and was associated with a marked increase in both Cystatin C and urine NGAL levels (Table 2). While death was relatively infrequent in the study population, there was a substantial increase in mortality among children with hyperphosphatemia and vitamin D deficiency. Vitamin D deficiency was not seen in children <5 years of age but increased to 12.6% in children ≥10 years of age and was more frequent in the context of structural kidney injury (such as children who were biomarker positive for NGAL).

We demonstrated that FGF23 and osteopontin were elevated in sMBD and strongly associated with mortality. FGF23 is a hormone excreted by osteoblasts and osteoclasts and regulates serum phosphate through the FGF receptor /Klotho complex in the kidneys (12). FGF23 has been found to predict mortality in patients with AKI following cardiac surgery (42), critically ill patients (43, 44), and patients with CKD (45). There is limited evidence that FGF23 may predict SCA-related mortality in adults (46), and our study provides additional evidence that increased FGF23 is associated with mortality in children with SCA. FGF23 reduces circulating plasma phosphate by decreasing renal reabsorption of phosphate and by blocking vitamin D activation. There was an 18-fold increase in median FGF23 levels in children with vitamin D deficiency and a negative correlation between FGF23 and 25-hydroxyvitamin D. However, we did not have measures of active 1,25 dihydroxy vitamin D to assess whether elevated FGF23 was inversely correlated with activated vitamin D. Osteopontin is a glycoprotein expressed mostly by the bones and in varying amounts from other cells such as macrophages, smooth muscle, endothelial and epithelial cells and is involved in inhibiting bone mineralization (10, 11). Studies among patients with sepsis (47) and critical illness (48, 49) reported elevated osteopontin among patients with a fatal outcome although there is limited data on the role of osteopontin in patients with SCA. Osteopontin is upregulated in vasculature in the setting of ischemia, and may also be released from renal tubular cells during acute kidney injury which can influence acute lung injury in mice (50–52). With findings from our study that highlight the role of both FGF23 and osteopontin in predicting mortality in SCA, there is need for further research to understand the interplay between bone biomarkers and kidney function in predicting mortality in children during a VOC.

Our study had several strengths including the comprehensive evaluation of sMBD parameters (calcium, phosphate, PTH, vitamin D, FGF23) in a relatively large cohort of children with SCA alongside a comprehensive assessment of kidney function (23, 24, 53). While the study was underpowered to assess biomarkers of mortality as there were only six deaths in the study population, the data were consistent with bone biomarkers being elevated in children who died while experiencing an acute pain crisis requiring hospitalization. A limitation of the study was that bone biopsies and bone density assessments were not conducted due to resource constraints (15). Additional studies are needed to evaluate serum biochemical abnormalities in conjunction with biopsy findings and to delineate the dynamics of biochemical markers of sMBD in children over clinical recovery, as well as regarding long term bone changes in relation to child growth.

In summary, mineral bone abnormalities were common in children with SCA hospitalized with vaso-occlusion and were associated with the presence of kidney disease and increased with age. As children with SCA experience frequent VOC that can accelerate the development of chronic bone disease over childhood (53), routine evaluation of bone mineral disorders is warranted. Osteopontin is a biomarker of bone disease or of acute ischemic injury and is associated with sMBD and mortality and may represent a novel biomarker to assess bone health or predict mortality in SCA. Finally, this study presents critical insight into sMBD in children with SCA in Africa, where the majority of people living with SCA reside but for which there are limited data. Recommendations regarding the appropriate management of mineral bone disorders remain problematic because they differ based on location, resource availability, the context in which it occurs and is further complicated in pediatric populations with unique needs across development. Based on the early age of sMBD onset in this cohort, we recommend policies promoting earlier assessment of kidney and bone health in this vulnerable population and the development of context-specific guidelines to support evidence-based care.

## Data Availability

The raw data supporting the conclusions of this article will be made available by the authors, without undue reservation.

## References

[1] Rees DC Williams TN Gladwin MT. Sickle-cell disease. Lancet. (2010) 376(9757):2018–31. 10.1016/S0140-6736(10)61029-X 21131035

[2] Piel FB Steinberg MH Rees DC. Sickle cell disease. N Engl J Med. (2017) 376(16):1561–73. 10.1056/NEJMra1510865 28423290

[3] Almeida A Roberts I. Bone involvement in sickle cell disease. Br J Haematol. (2005) 129(4):482–90. 10.1111/j.1365-2141.2005.05476.x 15877730

[4] Baxter-Jones AD Faulkner RA Forwood MR Mirwald RL Bailey DA. Bone mineral accrual from 8 to 30 years of age: an estimation of peak bone mass. J Bone Miner Res. (2011) 26(8):1729–39. 10.1002/jbmr.412 21520276

[5] Bailey DA McKay HA Mirwald RL Crocker PR Faulkner RA. A six-year longitudinal study of the relationship of physical activity to bone mineral accrual in growing children: the university of Saskatchewan bone mineral accrual study. J Bone Miner Res. (1999) 14(10):1672–9. 10.1359/jbmr.1999.14.10.1672 10491214

[6] Nih Consensus Development Panel on Osteoporosis Prevention D, Therapy. Osteoporosis prevention, diagnosis, and therapy. Jama. (2001) 285(6):785–95. 10.1001/jama.285.6.785 11176917

[7] Rovner AJ Stallings VA Kawchak DA Schall JI Ohene-Frempong K Zemel BS. High risk of vitamin D deficiency in children with sickle cell disease. J Am Diet Assoc. (2008) 108(9):1512–6. 10.1016/j.jada.2008.06.433 18755325

[8] Oladipo OO Temiye EO Ezeaka VC Obomanu P. Serum magnesium, phosphate and calcium in Nigerian children with sickle cell disease. West Afr J Med. (2005) 24(2):120–3. 10.4314/wajm.v24i2.28180 16092311

[9] Mohammed S Addae S Suleiman S Serum calcium, parathyroid hormone, and vitamin D status in children and young adults with sickle cell disease. Ann Clin Biochem. (1993) 30(Pt 1):45–51. 10.1177/000456329303000108 8382020

[10] Beck GR Jr Zerler B Moran E. Phosphate is a specific signal for induction of osteopontin gene expression. Proc Natl Acad Sci USA. (2000) 97(15):8352–7. 10.1073/pnas.140021997 10890885 PMC26951

[11] Mazzali M Kipari T Ophascharoensuk V Wesson J Johnson R Hughes J. Osteopontin—a molecule for all seasons. QJM. (2002) 95(1):3–13. 10.1093/qjmed/95.1.3 11834767

[12] Jüppner H. Phosphate and FGF-23. Kidney Int. (2011) 79:S24–7. 10.1038/ki.2011.27 26746858

[13] Batte A Menon S Ssenkusu JM Acute kidney injury in hospitalized children with sickle cell anemia. BMC Nephrol. (2022) 23(1):110. 10.1186/s12882-022-02731-9 35303803 PMC8933904

[14] Yee MM Jabbar SF Osunkwo I Chronic kidney disease and albuminuria in children with sickle cell disease. Clin J Am Soc Nephrol. (2011) 6(11):2628–33. 10.2215/CJN.01600211 21940843 PMC3359567

[15] Wheeler DC Winkelmayer WC. KDIGO 2017 Clinical practice guideline update for the diagnosis, evaluation, prevention, and treatment of chronic kidney disease-mineral and bone disorder (CKD-MBD) foreword. Kidney Int Suppl (2011). (2017) 7(1):1–59. 10.1016/j.kisu.2017.04.001 30675420 PMC6340919

[16] Hegde A Denburg MR Glenn DA. Acute kidney injury and pediatric bone health. Front Pediatr. (2020) 8:635628. 10.3389/fped.2020.635628 33634055 PMC7900149

[17] Yang T Wang W Tang X Association between mineral and bone disorder in patients with acute kidney injury following cardiac surgery and adverse outcomes. BMC Nephrol. (2019) 20(1):1–9. 10.1186/s12882-018-1181-1 31615432 PMC6794865

[18] Akrawinthawong K Shaw MK Kachner J Urine catalytic iron and neutrophil gelatinase-associated lipocalin as companion early markers of acute kidney injury after cardiac surgery: a prospective pilot study. Cardiorenal Med. (2013) 3(1):7–16. 10.1159/000346815 23946721 PMC3743453

[19] Kish L. Survey sampling. New York, USA: John Wiley and sons*.* (1965): 35–70.

[20] Pottel H Hoste L Martens F. A simple height-independent equation for estimating glomerular filtration rate in children. Pediatr Nephrol. (2012) 27(6):973–9. 10.1007/s00467-011-2081-9 22252520

[21] Batte A Starr MC Schwaderer AL Methods to estimate baseline creatinine and define acute kidney injury in lean Ugandan children with severe malaria: a prospective cohort study. BMC Nephrol. (2020) 21(1):417. 10.1186/s12882-020-02076-1 32993548 PMC7526147

[22] Nath KA Hebbel RP. Sickle cell disease: renal manifestations and mechanisms. Nat Rev Nephrol. (2015) 11(3):161–71. 10.1038/nrneph.2015.8 25668001 PMC4701210

[23] Martensson J Martling CR Bell M. Novel biomarkers of acute kidney injury and failure: clinical applicability. Br J Anaesth. (2012) 109(6):843–50. 10.1093/bja/aes357 23048068

[24] Greenberg JH Parikh CR. Biomarkers for diagnosis and prognosis of AKI in children: one size does not fit all. Clin J Am Soc Nephrol. (2017) 12(9):1551–7. 10.2215/CJN.12851216 28667085 PMC5586584

[25] Ostermann M Zarbock A Goldstein S Recommendations on acute kidney injury biomarkers from the acute disease quality initiative consensus conference: a consensus statement. JAMA Netw Open. (2020) 3(10):e2019209. 10.1001/jamanetworkopen.2020.19209 33021646

[26] Stanski N Menon S Goldstein SL Basu RK. Integration of urinary neutrophil gelatinase-associated lipocalin with serum creatinine delineates acute kidney injury phenotypes in critically ill children. J Crit Care. (2019) 53:1–7. 10.1016/j.jcrc.2019.05.017 31174170

[27] Pierce CB Muñoz A Ng DK Warady BA Furth SL Schwartz GJ. Age- and sex-dependent clinical equations to estimate glomerular filtration rates in children and young adults with chronic kidney disease. Kidney Int. (2021) 99(4):948–56. 10.1016/j.kint.2020.10.047 33301749 PMC9083470

[28] KDIGO. KDIGO clinical practice guideline for acute kidney injury. Kidney Int. (2012) 2(1):124–38. 10.1038/kisup.2012.1

[29] Pagana KD Pagana TJ Pagana TN. Mosby's diagnostic and laboratory tests. Fifteenth edn. Elsevier (2021).

[30] Colantonio DA Kyriakopoulou L Chan MK Closing the gaps in pediatric laboratory reference intervals: a CALIPER database of 40 biochemical markers in a healthy and multiethnic population of children. Clin Chem. (2012) 58(5):854–68. 10.1373/clinchem.2011.177741 22371482

[31] KDIGO. KDIGO 2012 Clinical practice guideline for the evaluation and management of chronic kidney disease. Kidney Int (Supplement). (2013) 3(1):1–150. 10.1038/kisup.2012.73 23989362

[32] Munns CF Shaw N Kiely M Global consensus recommendations on prevention and management of nutritional rickets. J Clin Endocrinol Metab. (2016) 101(2):394–415. 10.1210/jc.2015-2175 26745253 PMC4880117

[33] Wesseling-Perry K Salusky IB. Chronic kidney disease: mineral and bone disorder in children. Elsevier. (2013):169–79. 10.1016/j.semnephrol.2012.12.017 PMC4209124 23465503

[34] Audard V Moutereau S Vandemelebrouck G First evidence of subclinical renal tubular injury during sickle-cell crisis. Orphanet J Rare Dis. (2014) 9(1):67. 10.1186/1750-1172-9-67 24779676 PMC4006801

[35] Mogire RM Morovat A Muriuki JM Prevalence and predictors of vitamin D deficiency in young African children. BMC Med. (2021) 19(1):115. 10.1186/s12916-021-01985-8 34011341 PMC8136043

[36] Etzion Z Tiffert T Bookchin RM Lew VL. Effects of deoxygenation on active and passive Ca2 + transport and on the cytoplasmic Ca2+levels of sickle cell anemia red cells. J Clin Invest. (1993) 92(5):2489–98. 10.1172/JCI116857 8227363 PMC288434

[37] Goyal R Jialal I. Hyperphosphatemia. In: editors. Statpearls. Treasure island (FL): StatPearls Publishing (2022).

[38] Raj VM Freundlich M Hamideh D Abnormalities in renal tubular phosphate handling in children with sickle cell disease. Pediatr Blood Cancer. (2014) 61(12):2267–70. 10.1002/pbc.25188 25132581

[39] Sharpe CC Thein SL. Sickle cell nephropathy—a practical approach. Br J Haematol. (2011) 155(3):287–97. 10.1111/j.1365-2141.2011.08853.x 21902687

[40] Krishnamoorthy P Alyaarubi S Abish S Gale M Albuquerque P Jabado N. Primary hyperparathyroidism mimicking vaso-occlusive crises in sickle cell disease. Pediatrics. (2006) 118(2):e537–9. 10.1542/peds.2006-0337 16882790

[41] Denoix E Bomahou C Clavier L Primary hyperparathyroidism in sickle cell disease: an unknown complication of the disease in adulthood. J Clin Med. (2020) 9(2):308. 10.3390/jcm9020308 31979085 PMC7073651

[42] Leaf DE Christov M Juppner H Fibroblast growth factor 23 levels are elevated and associated with severe acute kidney injury and death following cardiac surgery. Kidney Int. (2016) 89(4):939–48. 10.1016/j.kint.2015.12.035 26924052 PMC4801748

[43] Rygasiewicz K Hryszko T Siemiatkowski A Brzosko S Rydzewska-Rosolowska A Naumnik B. C-terminal and intact FGF23 in critical illness and their associations with acute kidney injury and in-hospital mortality. Cytokine. (2018) 103:15–9. 10.1016/j.cyto.2017.12.024 29288982

[44] Leaf DE Jacob KA Srivastava A Fibroblast growth factor 23 levels associate with AKI and death in critical illness. J Am Soc Nephrol. (2017) 28(6):1877–85. 10.1681/ASN.2016080836 28028134 PMC5461795

[45] Isakova T Xie H Yang W Fibroblast growth factor 23 and risks of mortality and end-stage renal disease in patients with chronic kidney disease. Jama. (2011) 305(23):2432–9. 10.1001/jama.2011.826 21673295 PMC3124770

[46] Liu C-H Laja O Mendelsohn L Plasma FGF23 is a biomarker for left ventricular hypertrophy and mortality in adults with sickle cell anemia chronic kidney disease. Blood. (2015) 126(23):2171. 10.1182/blood.V126.23.2171.217126542250

[47] Carbone F Bonaventura A Vecchie A Early osteopontin levels predict mortality in patients with septic shock. Eur J Intern Med. (2020) 78:113–20. 10.1016/j.ejim.2020.04.035 32409206

[48] Roderburg C Benz F Cardenas DV Persistently elevated osteopontin serum levels predict mortality in critically ill patients. Crit Care. (2015) 19(1):271. 10.1186/s13054-015-0988-4 26111529 PMC4490692

[49] Lorenzen JM Hafer C Faulhaber-Walter R Osteopontin predicts survival in critically ill patients with acute kidney injury. Nephrol Dial Transplant. (2011) 26(2):531–7. 10.1093/ndt/gfq498 20732925

[50] Cen C Aziz M Yang WL Nicastro JM Coppa GF Wang P. Osteopontin blockade attenuates renal injury after ischemia reperfusion by inhibiting NK cell infiltration. Shock. (2017) 47(1):52–60. 10.1097/SHK.0000000000000721 27504800 PMC5167622

[51] Lok ZSY Lyle AN. Osteopontin in vascular disease: friend or foe? Arterioscler, Thromb, Vasc Biol. (2019) 39(4):613–22. 10.1161/ATVBAHA.118.311577 30727754 PMC6436981

[52] Khamissi FZ Ning L Kefaloyianni E Identification of kidney injury–released circulating osteopontin as causal agent of respiratory failure. Sci Adv. (2022) 8(8):eabm5900. 10.1126/sciadv.abm5900 35213222 PMC8880785

[53] Coca SG Yalavarthy R Concato J Parikh CR. Biomarkers for the diagnosis and risk stratification of acute kidney injury: a systematic review. Kidney Int. (2008) 73(9):1008–16. 10.1038/sj.ki.5002729 18094679

